# Influenza swine flu virus: A candidate for the next pandemic?

**DOI:** 10.7189/jogh.11.03011

**Published:** 2021-01-16

**Authors:** Mahnoor Yousif Shaikh, Farah Yasmin, Rohan Kumar Ochani, Syed Muhammad Ismail Shah

**Affiliations:** 1 Department of Internal Medicine, Dow University of Health Sciences, Karachi, Pakistan; 2 Department of Internal Medicine, Ziauddin Medical University, Karachi, Pakistan

A rising number of pneumonia cases were being reported in Wuhan, China since December 2019. Upon further investigation, it was discovered that the cause of these cases was a novel strain of the severe acute respiratory syndrome coronavirus-2 (SARS-COV-2). Shortly after, the World Health Organization (WHO) declared it a public health emergency of international concern (PHEIC) by January 2020 [1] and consequently, it was declared a pandemic two months later, affecting millions of lives worldwide. As of July 2, 2020, approximately 11 million people have been infected with this disease; with over half a million succumbed to the disease. Coronavirus disease-19 (COVID-19) is a respiratory illness that mainly spreads through airborne droplets via coughing or sneezing [2]. The disease severity ranges from infected individuals being asymptomatic to having life-threatening complications such as myocarditis, acute cerebrovascular disease, deep venous thrombosis, ischemic stroke, and pulmonary embolism [3]. It is due to these characteristics, in addition to the unavailability of the vaccine, and the rapid human-to-human transmission rate that traditional measures of combatting the virus were and are still being employed [4].

While the research scientists are still involved in devising an appropriate treatment for the SARS-CoV-2 disease, they are now concerned about another possible infectious outbreak that has an influenza pandemic potential that may further burden the already struggling health care system globally. Very recently, on June 30, 2020, a new strain of influenza swine flu virus named genotype 4 (G4) reassortant Eurasian avian-like (EA) H1N1 (G4 EA H1N1) virus was discovered by researchers in China. A recently published study in the journal of Proceedings of the National Academy of Sciences was based on the influenza virus surveillance of pigs across 10 Chinese provinces between 2011 and 2018. The findings of this study revealed that 10.4% of the swine workers and 4.4% of the general population exposed to the infected pigs tested positive for antibodies to G4 EA H1N1, virus and a higher seropositive rate of 20.5% was observed among those aged 18-35 years during the last three years of the study thus indicating that the virus has acquired increased human infectivity.

The researchers concluded that further mutations in the G4 virus may enhance their adaptation in humans coupled with their widespread circulation in the pig farms that may facilitate their exposure and hence, human-to-human transmission leading to a pandemic. Further research indicated that this G4 strain contains DNA from the 2009’s H1N1 strain, responsible for causing the pandemic that year in addition to the triple-reassortant (TR) derived internal genes. These viruses bind with high affinity to the human-like SAα2, 6Gal receptors, a prerequisite for infecting human cells. Additionally, G4 EA reassortant viruses replicate efficiently to produce much higher progeny viruses in the human airway epithelial cells including the human bronchial epithelial (NHBE) cells and alveolar epithelial (A549) cells, the primary target cells in human influenza virus infection. Finally, these viruses showed increased replication and pathogenicity in ferrets suggesting severe infection coupled with high virus transmission among ferrets both via direct contact (DC) and respiratory droplets (RD) exhibiting their capability to readily infect humans [5].

Scientists strongly recommend that the virus should be quickly controlled within the pigs and the human population, particularly workers in the swine industry, should be kept under surveillance [5]. Measures to contain this emerging virus must be taken immediately as it is believed that people may have little to no immunity to it and the current influenza flu vaccine does not offer protection against this strain. Furthermore, as most countries have started opening up for business despite the ongoing coronavirus pandemic, this new virus could also spread swiftly. Furthermore, health authorities have already been battling with an overwhelming number of SARS-CoV-2 cases and it has overburdened the medical system worldwide. Health care authorities and pharmaceutical companies are engaged in several collaborated and accelerated efforts to devise an efficient vaccine and management options to curtail the COVID-19 outbreak and combat the disease. Additionally, health care workers are already overwhelmed as they carry the highest risk of infection due to close contact with COVID-19 patients thus paralyzing health care systems [6].

Moreover, the lockdown, disruption in normal activities along with the uncertainty has placed considerable psychological stress in the general population, especially with anxiety and depression [7]. Another pandemic will lead to a further spike in mental health disorders which can, in turn, lead to adverse outcomes such as self-harm or suicide. Likewise, the global economy will suffer immensely in lieu of the coronavirus pandemic as International Monetary Fund (IMF) predicts that it will shrink by 3% this year, which is worse than the Great Depression seen in the 1930s [8]. Therefore, another pandemic will have a substantial impact on the global economy which can have disastrous consequences such as famine, drought, poverty, and even war.

**Figure:**
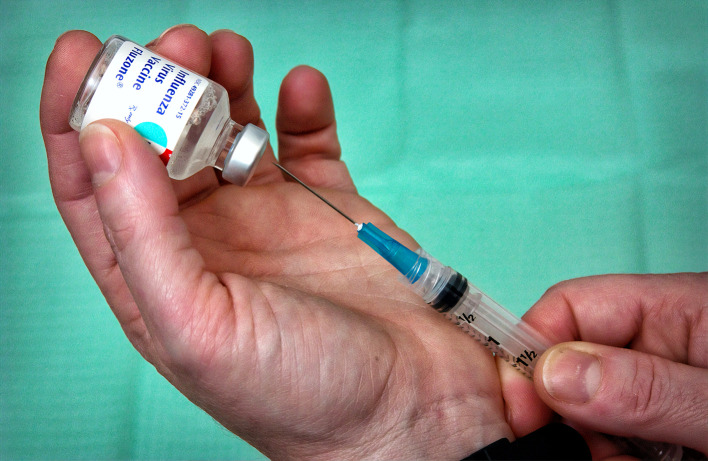
Photo: From https://unsplash.com/photos/_zFRhU7jqzc.

In conclusion, even amid the COVID-19 pandemic, scientists need to stay vigilant. Now the goal is to ensure that the G4 EA H1N1virus does not infect humans. A contingency plan must be formed and put in place in hospitals to not collapse an already overwhelmed health care system. Most importantly, the situation should be closely followed so that adequate measures, such as adapting the flu vaccine to the virus, can be taken immediately. World leaders must also work in conjunction with scientists and health care workers in taking effective measures to not just contain but combat any potential viruses. Furthermore, the media industry must also work towards providing authentic information verified by credible sources to create awareness and avoid public hysteria. Perhaps, the lessons learned from the COVID-19 pandemic can be applied here by containing the virus in its initial stages to prevent losing more lives.
